# Abdominal viscus penetration by laparoscopic-adjustable gastric band tubing: case report and review

**DOI:** 10.1259/bjrcr.20170097

**Published:** 2018-05-24

**Authors:** Francis T Delaney, Robert Franz

**Affiliations:** 1 Department of Medical Imaging, The Prince Charles Hospital, Brisbane, QLD, Australia; 2 Department of General Surgery, The Prince Charles Hospital, Brisbane, QLD, Australia

## Abstract

Laparoscopic-adjustable gastric band (LAGB) complications are increasingly recognised as follow-up time increases. These are most commonly related to the gastric band or port site, but complications of the connecting tubing are also reported. We present a case of LAGB tubing penetration through the transverse colon causing abdominal sepsis in a complex surgical abdomen and review prior published cases of abdominal viscus penetration by LAGB tubing. Like complications involving all LAGB components, these often present with non-specific abdominal signs and symptoms and undergo abdominal CT as an early investigation. This makes knowledge of normal and pathological imaging features of LAGB components important in radiology practice.

## Introduction

Surgical management of obesity has proven superior to any non-surgical approach.^1^ Laparoscopic-adjustable gastric band (LAGB) was the most common surgical technique employed for two decades following its introduction in the early 1990s.^2^ The use of LAGB has declined in recent years as long-term data has revealed device related complications and high re-operation rates.^3^ These complications may relate to any of the LAGB components, and while the port/reservoir and band itself account for the majority, connecting tubing complications are increasingly reported as follow-up time increases. LAGB complications typically present with non-specific abdominal symptoms and signs meaning a CT scan of the abdomen is often an early investigation.^4^

## Case

**Figure 1.**
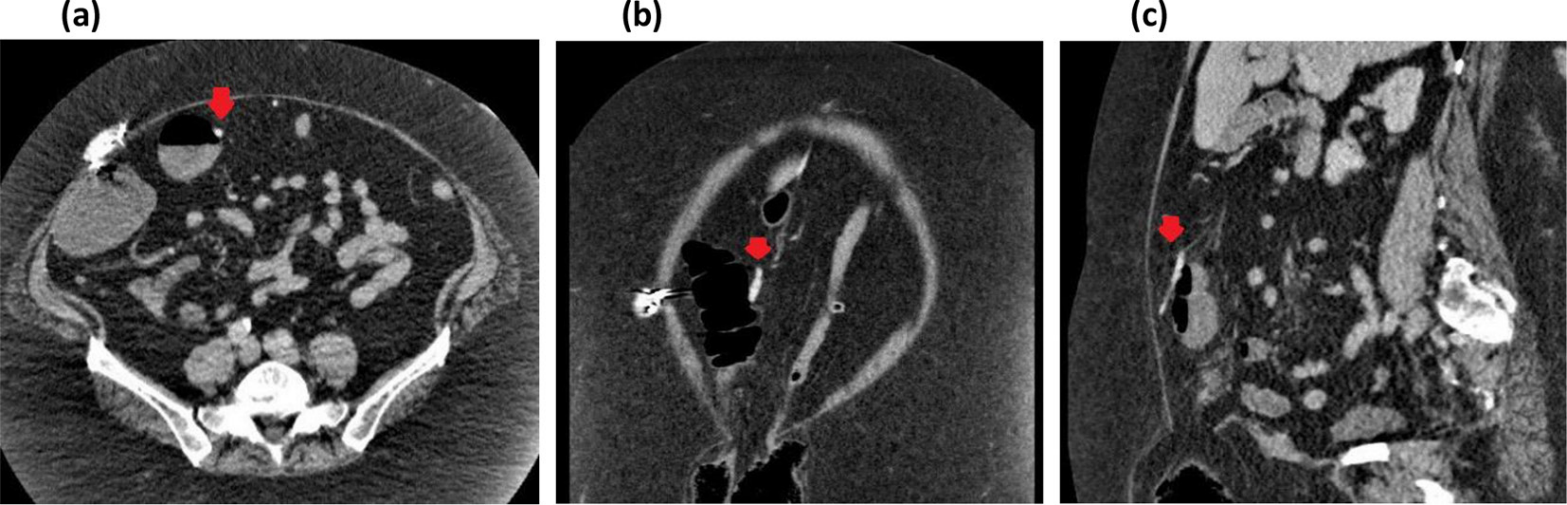
Axial (a), coronal (b) and sagittal (c) CT images showing the position of the laparoscopic-adjustable gastric band tubing (red arrow) adjacent to the transverse colon at time of initial presentation to our facility, 18 months prior to intracolonic penetration of the tubing.

We present the case of a 67-year-old female with a complicated surgical history. She initially presented to our facility with a strangulated large bowel containing ventral hernia, 10 years after undergoing LAGB procedure elsewhere. At this time, she remained morbidly obese and had been lost to follow-up. She underwent emergency surgical hernia repair with resection of necrotic transverse colon and loop ileostomy formation. One year later she presented with a LAGB port-site infection and had the port removed with the tubing sutured to the abdominal wall. During both admissions, the LAGB tubing was repeatedly seen in a stable position adjacent to the transverse colon on CT (Figure 1), with no concern for penetration.

**Figure 2.**
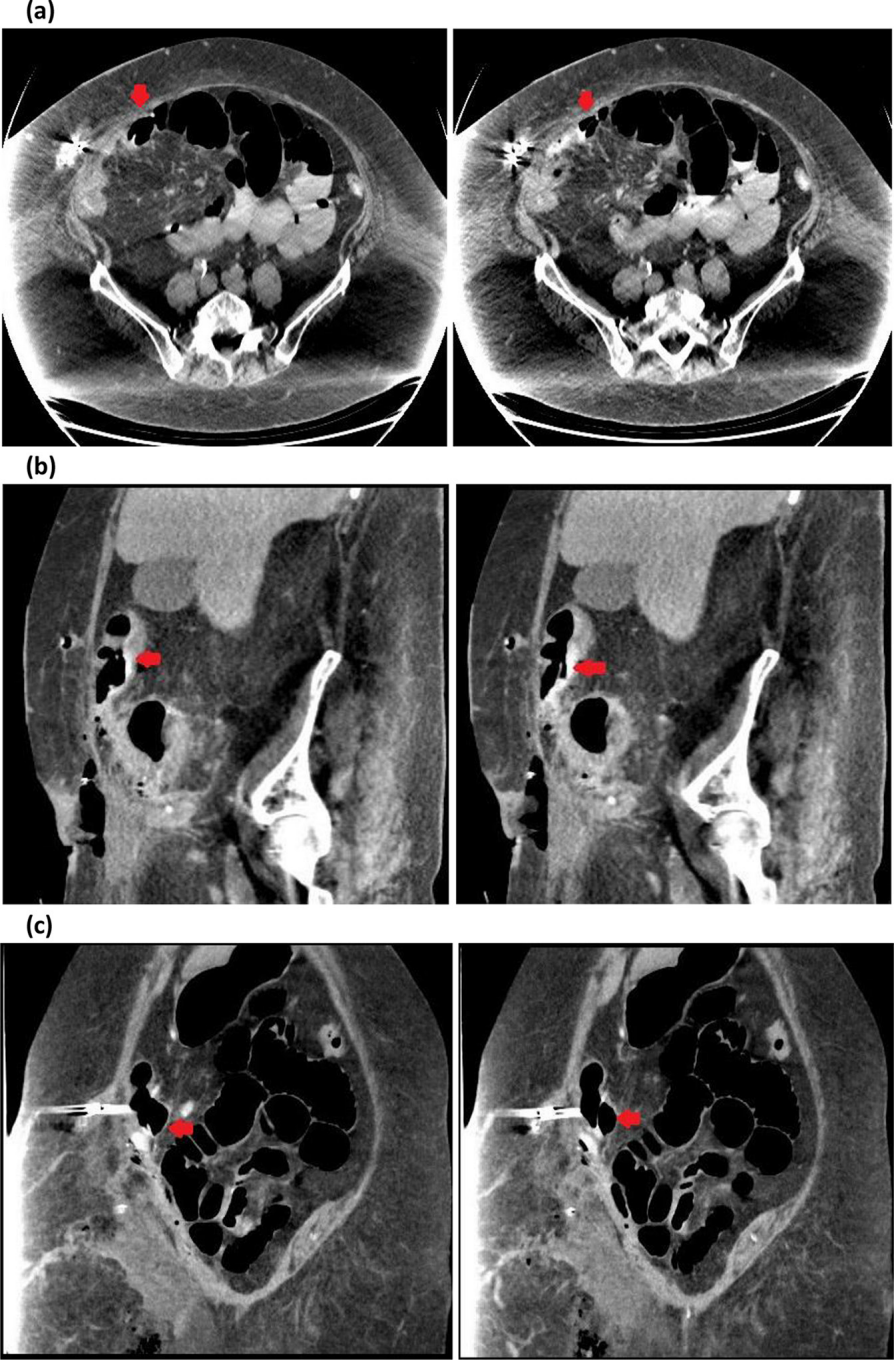
Axial (a), coronal (b) and sagittal (c) CT images showing laparoscopic-adjustable gastric band tubing (red arrow) penetrating through the transverse colon. The previous port site containing surgical clips can also be seen on axial images.

Elective reversal of the ileostomy was then performed 6 months following this but was complicated by abdominal sepsis in the early post-operative period. Abdominal CT demonstrated transection of the remaining proximal transverse colon by the LAGB tubing (Figure 2). This required right hemicolectomy with removal of gastric band and tubing and end ileostomy formation.

## Discussion

Intragastric migration/erosion of the band is a well-recognised LAGB complication reported to occur in 3.9% of cases, but penetration of the connecting tubing into an abdominal viscus is rare.^3^ Previously reported cases of LAGB tubing erosion into visceral structures are described in Table 1.^5–18^ Migration of different forms of intra-abdominal catheter into bowel wall has also been reported, and port system infections and the presence of a free end of tubing are predisposing factors.^5, 10^ Thus this case, in combination with previous literature, suggests that in patients in which the LAGB is no longer functioning intra-abdominal tubing should be removed when port-site removal or other abdominal surgery is performed. This is perhaps especially important when the tubing is seen on imaging to lie near a viscus which may be vulnerable to erosion over time. Awareness of this can help guide radiology reporting and surgical practice.

**Table 1.** Summary of previously reported cases of abdominal viscus erosion by LAGB tubing

Case	Eroded viscus	Imaging performed	CT findings	Definitive diagnosis	Prior port-site complication	Approximate time from LAGB to erosion
This case	Colon	CT	Tubing penetrating colon	CT	Infection and removal	11 years
Bell et al^5^	Colon	Intra-operative fluoroscopy	–	Laparoscopy with fluoroscopy	Infection and removal	3 years
Zengin et al^6^	Jejunum	–	–	–	–	–
Hartmann et al^7^	Colon	–	–	–	–	–
Navarra et al^8^	Colon	–	–	–	–	–
Mahtemework et al^9^	Jejunum	UGIE	–	Laparoscopy	No	3 years
Tekin^10^	Jejunum	None	–	Laparoscopy	Infection	
Povoa et al^11^	Colon	Colonoscopy, CT, UGIE	Tubing penetrating colon	CT	No	4 years
Tan et al^12^	Colon	AXR, CT, UGIE	Tubing looping around small bowel mesentery	Laparotomy	Dislocation and revision	5 years
Cintolo et al^13^	Duodenum	Barium study, CT, UGIE	Tubing penetrating duodenum	CT in retrospect after UGIE	Infection and removal	1 year
Pfeiffer et al^14^	Colon	None	–	Tubing visible per rectum	Infection and removal	6 months
Blouhos et al^15^	Colon	CT	Inflammation around tubing ending in inflammatory mass	Laparotomy	No	4 years
Alkhaffaf et al^16^	Jejunum	UGIE, CT	Tubing penetrating jejunum	CT	Infection and removal	5 years
Strahan et al^17^	Colon	CT	Tubing penetrating colon	CT	No	12 years
Sneijder et al^18^	Kidney	UGIE, CT	Tubing penetrating kidney	CT	Infection and removal	1.5 years

AXR, abdominal X-ray; LAGB, laparoscopic-adjustable gastric band; UGIE, upper gastrointestinal endoscopy.

Prior port-site complication – complication related to port site before presenting with tubing erosion.

Although the number of new LAGB procedures being performed is reducing steadily there are a large cohort of patients with this device already in place. As LAGB tubing complications are primarily related to mechanical stress they are likely time-dependent and prevalence will continue to increase with ongoing follow-up.^3, 10^

While plain radiographs and upper gastrointestinal series may be used to evaluate LAGB complications, most cases will undergo abdominal CT as part of investigation.^4, 19,20^ This may be diagnostic as in our case, but as demonstrated in Table 1, diagnosis can be difficult and may only be definitively made intra-operatively.

## Conclusions

Complications related to LAGB tubing are increasingly recognised in clinical practice. When combined with the ever-expanding use of CT for investigation of abdominal complaints, this highlights the importance of the imaging features of all LAGB components for radiologists. Clear visualisation of LAGB tubing penetration through the colon as demonstrated here has rarely been reported.

## Learning Points

1. LAGB complications can be related to any of the components including the connecting tubing, and tend to present with non-specific abdominal signs and symptoms and undergo abdominal CT
2. LAGB tubing complications often occur in patients who have had previous revision or removal of the LAGB port site due to infection
3. LAGB tubing has the potential to penetrate through abdominal structures and may cause severe illness
4. The large numbers of patients with LAGB devices in place coupled with the increase in tubing-related complications as follow-up time increases makes knowledge of the normal and abnormal imaging features of LAGB tubing important in radiology practice
